# An Uncommon Presentation of Sweet’s Syndrome in Primary Myelofibrosis

**DOI:** 10.1155/crh/4413111

**Published:** 2026-04-06

**Authors:** Valentina Rago, Valeria Brogna, Fabrizio Cavalca, Matteo Faltoni, Antonio Maria Alviano, Carlo Gambacorti-Passerini, Elena Maria Elli

**Affiliations:** ^1^ Department of Medicine and Surgery, University of Milano–Bicocca, Milano, Italy; ^2^ Department of Hematology and Bone Marrow Transplant Unit, San Gerardo dei Tintori IRCCS Foundation, Monza, Italy; ^3^ Department of Infectious Diseases, San Gerardo dei Tintori IRCCS Foundation, Monza, Italy; ^4^ Department of Pathology, San Gerardo dei Tintori IRCCS Foundation, Monza, Italy

**Keywords:** case report, corticosteroids, myelofibrosis, ruxolitinib, Sweet’s syndrome

## Abstract

Sweet’s syndrome (SS) is a rare neutrophilic dermatosis often associated with hematologic malignancies. Due to its infectious mimicry, diagnosis is frequently delayed. We report a case of primary myelofibrosis initially treated for suspected cellulitis, which progressed to necrotic lesions and refractory fever despite broad‐spectrum antibiotics. A skin biopsy confirmed neutrophilic vasculitis consistent with SS. Prompt treatment with corticosteroids followed by ruxolitinib achieved rapid resolution by suppressing the underlying cytokine storm.

## 1. Introduction

Here, we report an atypical presentation of an inflammatory skin disorder associated with primary myelofibrosis (MF). The patient experienced progressive clinical systemic deterioration associated with cutaneous manifestations secondary to Sweet’s syndrome (SS). This presentation required a multidisciplinary approach for diagnosis and management and ultimately resolved successfully.

## 2. Case Presentation

We present the case of a 66‐year‐old man under follow‐up at our hematology department for prefibrotic MF with a CALR mutation (52‐bp deletion) diagnosed in 2014. His medical and family history was essentially unremarkable. Initially, he had mild thrombocytosis without constitutional symptoms or significant splenomegaly; thus, hydroxyurea was started. In 2017, due to failure to control thrombocytosis and leukocytosis, treatment was switched to subcutaneous pegylated interferon. By 2021, he developed worsening leukocytosis (WBC 27,000/μL) with immature peripheral myeloid cells along with symptomatic splenomegaly. Ruxolitinib, a JAK2 inhibitor, was initiated based on an intermediate‐2 Dynamic International Prognostic Scoring System (DIPSS) score. In 2024, a bone marrow (BM) histological reassessment was performed due to constitutional symptoms (night sweats) and transfusion‐dependent anemia, revealing progression to overt MF. Next‐generation sequencing (NGS) showed a CALR mutation, along with mutations in the DNMT3A and SF3B1 genes. Cytogenetics revealed a complex karyotype. The patient was classified as having high DIPSS and DIPSS‐plus scores. Treatment was switched to fedratinib, a second‐generation JAK2 inhibitor, as a bridge to allogeneic stem cell transplantation. However, due to a suspected allergic reaction (laryngeal edema), the drug was discontinued, and hydroxyurea was reintroduced to control proliferative disease, along with red blood cell transfusions for anemia. The disease remained stable until February 15, 2025, when he presented to the emergency department with a 4 × 5 cm purulent cystic lesion on the right temporal side of the head accompanied by disseminated nonpruritic, erythematous papules on the limbs and low‐grade fever. Blood tests showed neutrophilic leukocytosis (a WBC count of 29,000/μL with 22,690 neutrophils without blasts), elevated C‐reactive protein (CRP), and worsening anemia (hemoglobin 8.9 g/dL), consistent with underlying chronic myeloproliferative disease and ongoing cytoreductive therapy. Procalcitonin (PCT), initially low, increased in the following days (up to 31 mcg/L, range: < 0.5 mcg/L). An initial suspicion of staphylococcal pustulosis led to empiric amoxicillin/clavulanic acid. Despite this, persistent low‐grade fever prompted outpatient reassessment with a single dose of dalbavancin.

A week later, due to clinical deterioration and worsening fever, the patient was admitted to the infectious diseases department for further evaluation and targeted treatment. Upon admission, a new erythematous, edematous, warm, and painful tender plaque of 15 × 10 cm was observed in the right pectoral area, raising suspicion of cellulitis. Empiric intravenous therapy with piperacillin/tazobactam, daptomycin, and clindamycin was started. However, the fever persisted, and skin lesions rapidly progressed from disseminated, erythematous, indurated plaques (Figures 1(a) and 2(a)) into ulcerated plaques on the limbs, trunk, and sacral region, increasing in size (Figure 1(b)). Due to the rapid skin and clinical deterioration with fever refractory to continuous infusion of paracetamol, liposomal amphotericin B was added for broad‐spectrum antifungal coverage. Despite these measures, the patient’s condition worsened further: leukocytosis reached 60,000/mm^3^, and Grade 3 anemia and thrombocytopenia appeared, leading to reduction and eventual discontinuation of hydroxyurea. Additionally, CRP increased up to 180 mg/L (range: 0–5 mg/L), and PCT levels remained elevated.

FIGURE 1Lesions at the level of the iliac crest at the onset: an erythematous, indurated plaque measuring 4 × 4 cm (a). Evolution of the lesion during the acute phase of the disease: an erythematous, indurated, ulcerated plaque (b) and in the resolving phase: an erythematous, scaly plaque (c).

**Figure:**
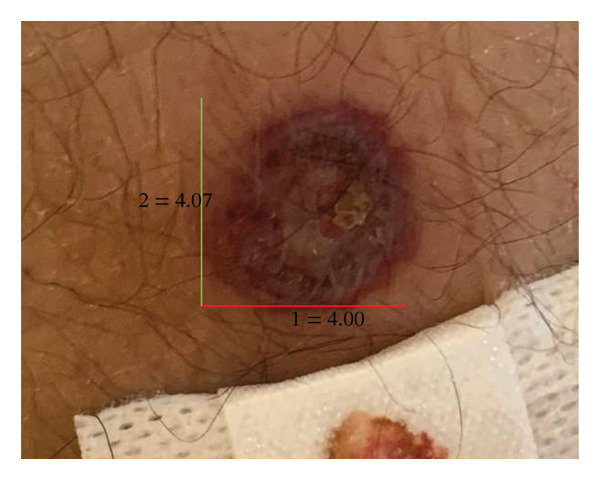
(a)

**Figure:**
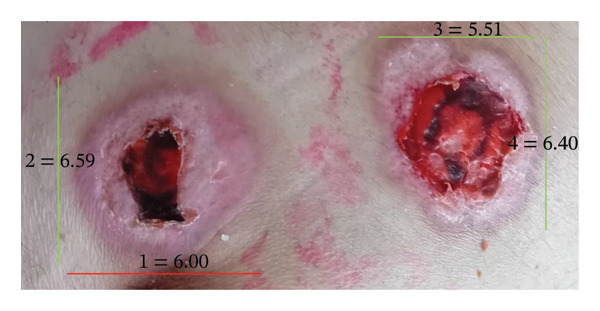
(b)

**Figure:**
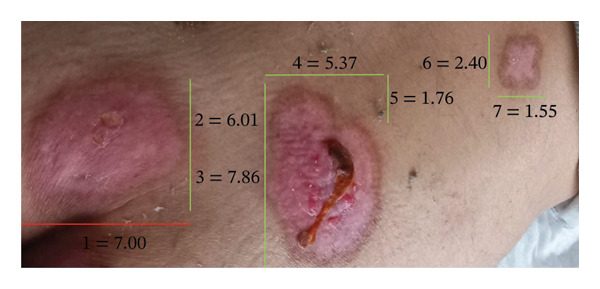
(c)

FIGURE 2Elbow lesion in the acute phase of the disease: an erythematous, indurated plaque measuring 6 × 7 cm (a) and in the resolving phase: an erythematous, scaly plaque measuring nearly 6 × 6 cm (b).

**Figure:**
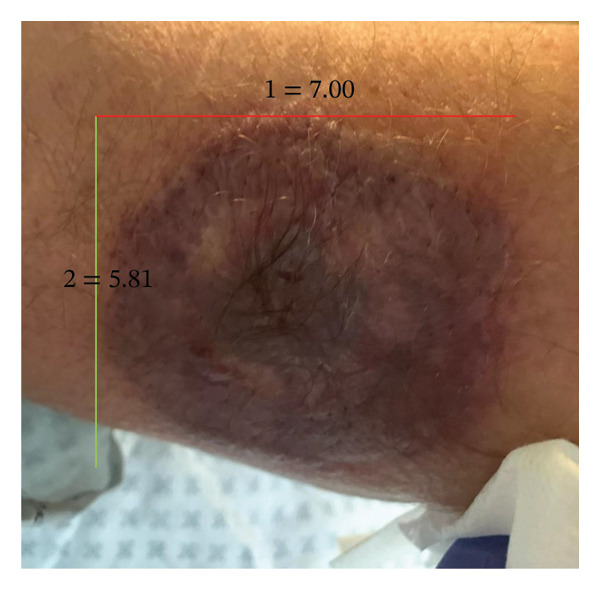
(a)

**Figure:**
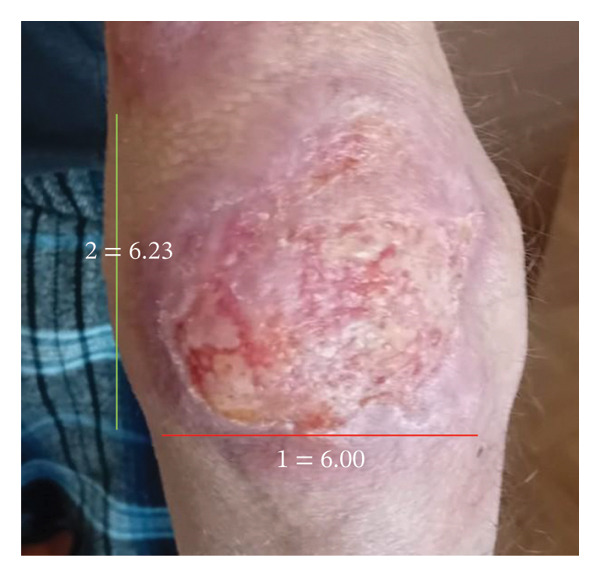
(b)

A comprehensive diagnostic workup was performed to explore possible differential diagnoses underlying the patient’s presentation:

• Infectious causes were ruled out through serial blood cultures during febrile episodes, wound swabs, and tests for serum β‐D‐glucan, galactomannan, cryptococcal antigen, Leishmania serology, and herpesvirus viremia—all negative. A total‐body CT scan showed no signs of organ involvement.
• Autoimmune causes were excluded with negative autoantibody panels (antinuclear antibodies [ANA], extractable nuclear antigen [ENA] antibodies, anti–double‐stranded DNA (anti‐dsDNA) antibodies, rheumatoid factor (RF), complement, antismooth muscle antibodies (ASMA), and antimitochondrial antibodies).
• BM aspiration ruled out progression to acute leukemia.

With the growing suspicion of an inflammatory disorder associated with the known underlying chronic hematological disease, dermatology was consulted for a punch biopsy of one of his skin lesions. The skin biopsy showed a diffuse dermal infiltrate of neutrophils and scattered histiocytes in a vaguely nodular pattern, with intraepidermal exocytosis, marked dermal edema, and localized epidermal erosion. Periadnexal and perivascular involvement by the neutrophilic infiltrate was noted, along with neutrophil karyorrhexis, endothelial cell swelling, and vascular congestion. No evidence of immature myeloid elements (CD34+) was found (Figure 3). The differential diagnosis included SS and pyoderma gangrenosum (PG). However, the limited epidermal erosion and the presence of karyorrhectic debris were more in favor of the former diagnosis. All clinical, histological, and laboratory criteria of SS, according to Driesch and subsequent revisions, were met [1].

FIGURE 3Diffuse neutrophilic infiltrate involving the dermis and dermo–epidermal junction, with a vaguely nodular appearance at low power (a). At higher magnification, marked dermal edema is evident, along with abundant neutrophils and mononuclear elements (b), intraepidermal neutrophil exocytosis (c), and localized epidermal erosion (d). Periadnexal (e) and perivascular (f) involvement by the dermal infiltrate is also noted, with slight vascular congestion and endothelial cell swelling (g). The dermal infiltrate comprises scattered histiocytes, as highlighted by CD68 immunohistochemistry (h).

**Figure:**
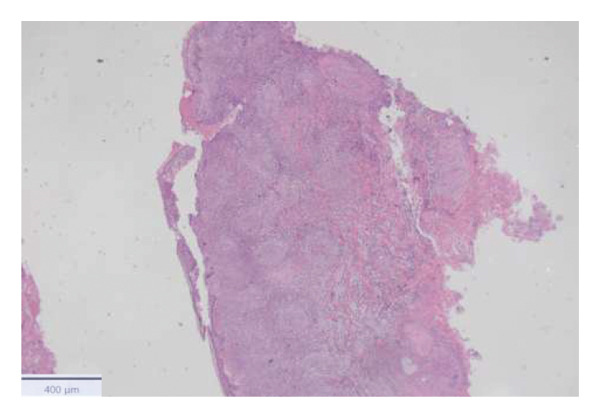
(a)

**Figure:**
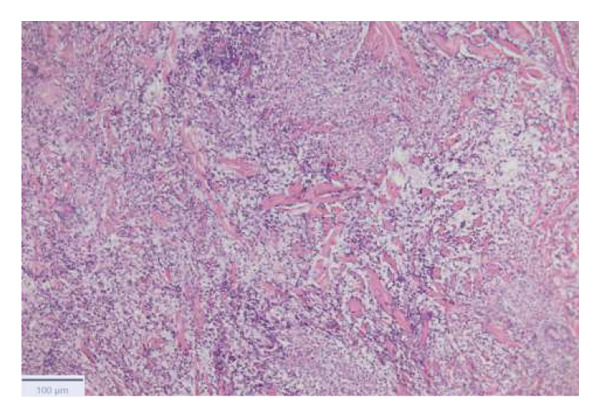
(b)

**Figure:**
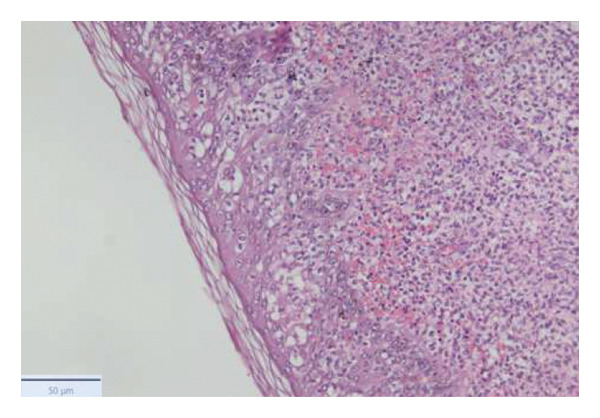
(c)

**Figure:**
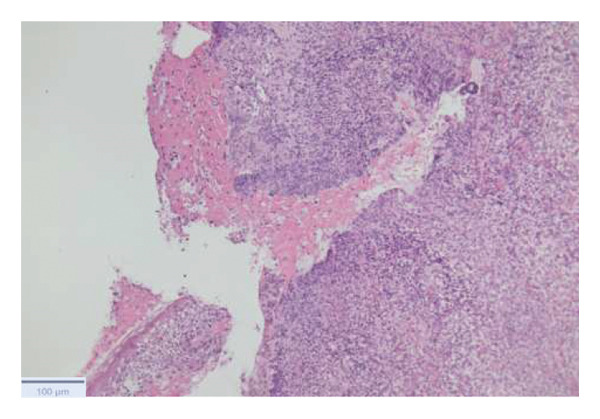
(d)

**Figure:**
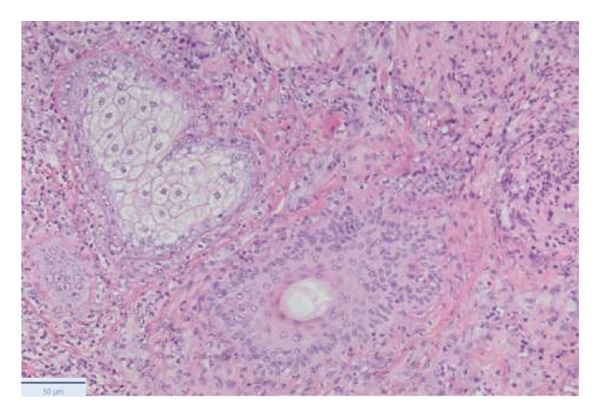
(e)

**Figure:**
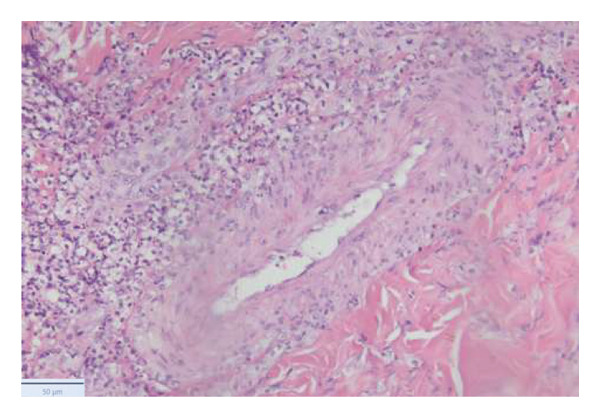
(f)

**Figure:**
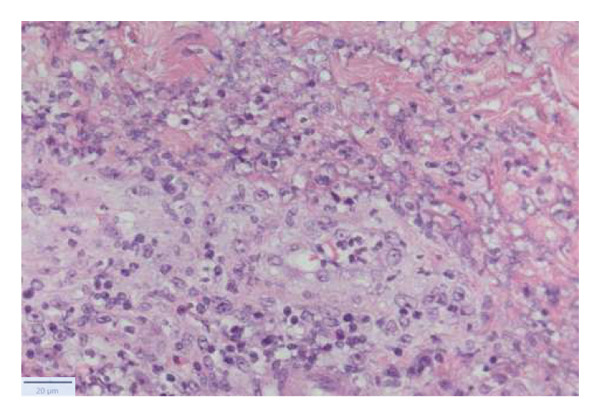
(g)

**Figure:**
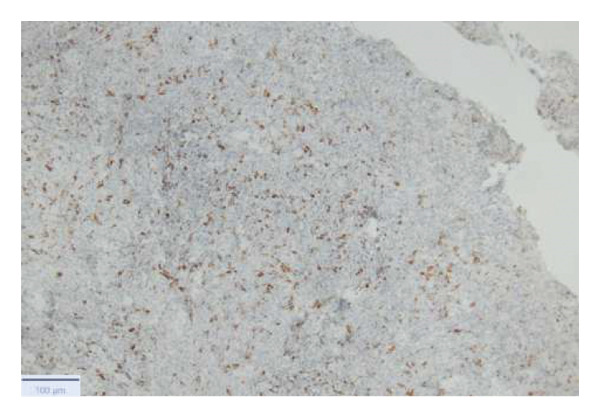
(h)

Corticosteroid therapy with 6‐methylprednisolone 1 mg/kg/day was promptly started on March 14, leading to rapid defervescence, significant reduction in the size of the skin lesions, normalization of inflammatory markers, and improvement in the blood counts. Five days later, ruxolitinib 5 mg twice daily was added for its JAK–STAT immunomodulatory effect to manage both the cytokine storm and the underlying hematologic disease. This combined regimen enabled steroid tapering while maintaining clinical stability and hematologic improvement, leading to complete resolution and discharge on April 2. One month postdischarge, the patient demonstrated further recovery with a 4.5‐kg weight gain and resolving skin lesions (Figures 1(c) and 2(b)). Blood count normalization allowed for increasing ruxolitinib to 10 mg twice daily to better manage the hematologic condition (Table 1). No significant adverse effects related to immunosuppressive treatment were reported.

**TABLE 1:** Case report timeline.

Date	Clinical presentation	Treatment
February, 15, 2025	Fever, onset of a 4 × 5 cm suppurative cystic lesion on the right temporal side of the head accompanied by disseminated nonpruritic, erythematous papules on the limbs	Broad‐spectrum antibiotic
March, 1 2025	Clinical deterioration, worsening fever, a lesion resembling cellulitis in the pectoral area	Hospitalization, maximized antibiotic therapy, addition of antifungal therapy
March, 14 2025	Refractory fever, multiple diffuse ulcerated plaques with progressive worsening and cytopenias	6‐methylprednisolone 1 mg/kg/day
March, 19 2025	Slight initial improvement of skin lesions, disappearance of fever	Ruxolitinib 5 mg twice daily in addition to steroid therapy
April, 2 2025	Skin lesions in the resolving phase, normalization of inflammatory laboratory markers	Discharge with steroid tapering, continuation of ruxolitinib twice daily
May, 2 2025	Skin lesions in the resolving phase, hematological improvement	Ruxolitinib to 10 mg twice daily

*Note:* Presented according to the CARE guidelines.

## 3. Discussion

SS is classified among neutrophilic dermatoses, a group of inflammatory skin disorders. Although the etiology of this disorder is still largely unknown, it has been recognized that a cytokine storm underlies its pathogenesis, triggered by an abnormal reaction to a tumor, viral, or bacterial antigen [2, 3]. Based on the underlying etiology, three subtypes of SS are recognized: idiopathic, drug‐induced, and secondary to neoplastic diseases [4]. The latter is primarily associated with hematological malignancies, and it could be the initial manifestation or a sign of disease relapse. In our case, SS represented an uncommon manifestation of a paraneoplastic syndrome linked to MF in the chronic phase; the BM aspiration allowed us to exclude the progression to acute leukemia.

Very few cases of SS associated with MF have been reported from 1993 to the present (Table 2) [5], highlighting the rarity of this association.

**TABLE 2:** Summary of the case reports of published cases of MF and SS.

Year published, reference	Age/sex	Clinical features: skin lesion description	Treatment of SS	Outcome of SS
Case report	66 years/M	Fever, a suppurative cystic lesion, then a lesion resembling cellulitis, then multiple ulcerated plaques	Steroids and ruxolitinib	Complete resolution
Thebo et al. [5]	45 years/M	Fever, erythematous plaques on the left thigh, left groin, and umbilical region	Steroids	Complete resolution
Xiang et al. [6]	75 years/F	Papules and plaques on the trunk and extremities. On the lower leg, a painful, indurated, edematous plaque resembling cellulitis	Steroids	Complete resolution
Su et al. [7]	26 years/M	Plaques and nodules	Steroids	After tapering, rebound of SS > chemotherapy (cytosine arabinoside) and death
Chatterjee et al. [8]	77 years/F	Multiple pustular lesions, hand swelling	Steroids	Just little improvement > death
Jiang et al. [9]	77 years/M	Ileitis and skin bullae	Steroids	Complete resolution
Sakoda et al. [10]	59 years/M	Plaque on the right buttock with soft tissue infiltration, fever	Steroids	Complete resolution
Gowda et al. [11]	66 years/F	Leg wound	Steroids	Complete resolution
Che et al. [12]	70 years/M	Fever, erythema with painful nodules	Steroids	Complete resolution
Shiga et al. [13]	61 years/M	Fever, painful eruptions on the face and upper extremities	Steroids	Complete resolution
Brodkin and Schwarts [14]	68 years/M	Solitary enlarging lesions on the dorsum of the hand and face	Steroid	Initial improvement > death
Delgado et al. [15]	69 years/M	Fever, erythematous painful lesions on scalp, brow, and thigh	Steroid	Initial improvement > death

*Note:* M: male, F: female; y: years; MF: myelofibrosis.

Abbreviation: SS, Sweet’s syndrome.

In the MF setting, some drugs, including hydroxyurea [16], tyrosine kinase inhibitors [17, 18], or hypomethylating agents [19], have been reported to affect cytokine levels and thus be implicated in SS pathogenesis. Interestingly, some cases developed soon after starting or stopping ruxolitinib treatment [5], possibly due to incomplete cytokine suppression or cytokine rebound after discontinuation, respectively. In our case, the addition of low‐dose ruxolitinib to steroid therapy was primarily intended to achieve cytokine suppression rather than immediate hematologic control. Given the severe inflammatory state associated with SS and advanced MF, ruxolitinib was selected for its immunomodulatory effects on the JAK–STAT pathway. The low starting dose (5 mg twice daily) reflected the presence of active systemic inflammation and significant thrombocytopenia, aiming to limit hematologic toxicity. Hematologic disease control became a secondary goal and was addressed later through dose escalation once clinical stability and blood count recovery were achieved.

An increased incidence of SS has been hypothesized in patients with MF carrying somatic mutations commonly found in myelodysplastic syndromes (MDSs). Approximately 21% of patients with SS have an associated malignancy, and up to 80% of cases are associated with hematological diseases, predominantly MDS or acute myeloid leukemia [20]. Therefore, it has been hypothesized that the presence of MDS‐related mutations may predispose to the development of SS. However, further studies are required to confirm this association. Our case supports this hypothesis, since NGS analysis revealed the presence of somatic myeloid mutations in epigenetic (DNMT3A) or spliceosome (SF3B1) genes in addition to a CALR mutation.

The diagnosis of SS is defined by the presence of specific clinical, laboratory, and histopathological criteria [1]: The most common clinical presentation involves the appearance of rapidly progressing, disseminated skin lesions, associated with fever and elevated inflammatory markers. Histologically, dense neutrophilic infiltration in the reticular dermis is characteristic, along with marked papillary dermal edema. Usually, lesions appear on the arms, face, or neck, but atypical presentations can occur [5]. In our patient, initial skin lesions mimicked cellulitis, similar to the case reported by Thebo et al. [5], where cellulitis preceded SS, suggesting that skin changes can sometimes be the earliest sign of the disease.

In the absence of large clinical trials, the management of SS is primarily guided by expert consensus and case reports. Glucocorticoids are the first‐line therapy, with oral prednisone (0.5–1 mg/kg/day) typically producing rapid improvement in fever and skin lesions within 1–2 weeks.

Accurate differentiation between SS and infection is vital yet difficult in immunocompromised settings. Our patient’s presentation—fever, neutrophilia, elevated PCT, and a localized plaque—strongly mimicked cellulitis, leading to initial broad‐spectrum antibiotic treatment and highlighting the diagnostic pitfalls in neutrophilic dermatoses. However, the lack of microbiological evidence, poor response to antibiotics, multifocal skin involvement, and rapid progression of lesions were key clues to a noninfectious inflammatory process. Skin biopsy and the prompt response to steroid treatment were decisive in establishing the diagnosis of SS.

PG, another neutrophilic dermatosis, is often associated with hematologic diseases like MDS. A reported case linked to MF responded poorly to steroids alone but improved with adalimumab and ruxolitinib [21]. In contrast, our patient showed a prompt and robust steroid response. Furthermore, the clinical presentation and histopathologic features were more consistent with SS rather than PG. First, our patient did not have a history of pathergy. Second, the skin biopsy showed only limited epidermal erosion, along with neutrophil karyorrhexis, which is an uncommon microscopic finding in PG.

In conclusion, inflammatory hyperactivation in hematologic malignancies like MF can manifest with atypical clinical features and represents a diagnostic challenge. In our case, the evolution from cellulitis‐like symptoms to progressive skin involvement obscured the early recognition of a neutrophilic dermatosis. A multidisciplinary approach with early dermatologic consultation is essential to avoid diagnostic delays, reduce unnecessary antimicrobial therapy, and ensure timely immunosuppressive intervention.

## Author Contributions

Dr, Elena Maria Elli, Dr. Fabrizio Cavalca, and Dr. Valentina Rago (as resident) were responsible for the management of the patient’s hematological condition. Dr. Matteo Faltoni and Dr. Valeria Brogna (as resident) managed the patient during hospitalization, evaluating possible infectious differential diagnoses. Dr. Antonio Maria Alviano performed the histopathological analysis of the tissue sample. All authors, under the supervision of Prof. Carlo Gambacorti‐Passerini, contributed to the drafting and revision of the case report and approved the final version.

## Funding

The authors received no specific funding for the preparation of this case report. It is the result of the clinical activity carried out by all the authors as part of their professional practice. The authors intend to request APC coverage from the University of Milano‐Bicocca under the Wiley and CARE‐CRUI agreement.

## Disclosure

All authors have read and approved the final version of the manuscript. Corresponding author Dr. Valentina Rago had full access to all data in this study and takes complete responsibility for the integrity of the data and the accuracy of the data analysis. The corresponding author is affiliated with the University of Milano‐Bicocca.

## Ethics Statement

The patient provided written consent for the use of images for research and publication purposes. All details have been anonymized to ensure privacy.

## Conflicts of Interest

The authors declare no conflicts of interest.

## Data Availability

No datasets were generated or analyzed during the preparation of this case report.
